# Low-frequency passive mechanical stimulation produces antidepressant-like effects in rats involving gut–brain axis modulation

**DOI:** 10.3389/fimmu.2026.1928978

**Published:** 2026-10-12

**Authors:** Rong Gao, Wenjing Pu, Yifei Lv, Liqiong Liu, Yan Zhao, Hao Liu, Li Chen, Nan Guan, Hongen Li

**Affiliations:** 1 School of Basic Medicine, Gansu Medical College, Pingliang, China; 2 Key Laboratory of Microbial Cohort and Multi-omics Research in Gansu Province, Gansu Medical College, Pingliang, China; 3 Pingliang Mental Health Center, Pingliang, China; 4 School of Clinical Medicine, Gansu Medical College, Pingliang, China; 5 Affiliated Hospital of Gansu Medical College, Pingliang, China

**Keywords:** CUMS, depression, gut microbiota, metabolome, passive vertical oscillation

## Abstract

**Introduction:**

Depression is associated with dysregulation of neuroendocrine, immune, and gut -brain axis pathways. Exercise is an effective non-pharmacological intervention; however, poor motivation and fatigue limit adherence in patients with depression.

**Methods:**

We investigated a novel passive exercise modality, low-frequency passive vertical oscillation assisted by elastic ropes (LF-PVO-ER), in a chronic unpredictable mild stress (CUMS) rat model.

**Results:**

LF-PVO-ER significantly improved depressive-like behaviors, including anhedonia and behavioral despair. Alongside these behavioral improvements, increases were observed in serum serotonin and brain-derived neurotrophic factor (BDNF), along with reductions in serum corticosterone, inflammatory markers (IL-6, TNF), and lipopolysaccharide (LPS). Histological analysis revealed attenuation of hippocampal and colonic injury. 16S rRNA sequencing showed partial restoration of CUMS-induced gut microbiota dysbiosis, as reflected by increased diversity and enrichment of short-chain fatty acid (SCFA)-associated genera. Untargeted metabolomics revealed changes in amino acid, lipid, and bile acid metabolism. Integrated analyses indicated coordinated associations among gut microbiota, metabolites, and neuroimmune markers.

**Conclusion:**

These findings suggest that LF-PVO-ER may exert antidepressant-like effects through multiple pathways involving neuroendocrine function, inflammatory responses, and gut microbiota -metabolite interactions, supporting its potential as a passive exercise strategy for depression intervention.

## 1 Introduction

Depression is a prevalent and disabling psychiatric disorder characterized by persistent low mood, anhedonia, cognitive dysfunction, and somatic symptoms (1). Affecting more than 280 million people worldwide, depression is a leading cause of disability and contributes substantially to healthcare expenditure, reduced productivity, and diminished quality of life (2). Although its precise pathogenesis remains incompletely understood, depression is now recognized as a multifactorial disorder involving complex interactions among genetic susceptibility, environmental stressors, neuroendocrine dysregulation, impaired monoaminergic neurotransmission, reduced neuroplasticity, hypothalamic–pituitary–adrenal (HPA) axis hyperactivity, and chronic low-grade inflammation (3). Rather than resulting from dysfunction within the central nervous system alone, increasing evidence suggests that depression represents a systemic disorder involving bidirectional communication among the brain, immune system, endocrine pathways, and gastrointestinal tract.

Among these mechanisms, the gut–brain axis has emerged as a critical regulator of emotional behavior and stress resilience (4). The gut microbiota communicates with the central nervous system through multiple interconnected pathways, including neural signaling *via* the vagus nerve, endocrine regulation, immune modulation, and the production of microbial-derived metabolites (5). Chronic psychological stress can disrupt the composition and diversity of the gut microbiota, impair intestinal barrier integrity, and promote the translocation of bacterial products such as lipopolysaccharide (LPS) into the circulation (6). These alterations amplify systemic inflammation and contribute to neurotransmitter imbalance, microglial activation, and impaired synaptic plasticity, thereby promoting depressive-like behaviors (6). Conversely, restoration of microbial homeostasis through dietary interventions, probiotics, faecal microbiota transplantation, or physical exercise has demonstrated antidepressant-like effects in both experimental and clinical studies (7). In particular, microbial-derived short-chain fatty acids (SCFAs) have attracted considerable attention because of their roles in maintaining intestinal barrier integrity, regulating immune responses, and modulating neurotrophic signaling and neurotransmitter synthesis (8).

Regular physical exercise is widely recognized as one of the most effective non-pharmacological interventions for depression. In addition to improving mood and cognitive function, exercise enhances neuroplasticity by increasing brain-derived neurotrophic factor (BDNF), restores HPA axis homeostasis, suppresses chronic inflammation, and promotes favorable remodeling of the gut microbiota and host metabolism (9). Consequently, exercise is recommended as an important adjunct to pharmacological and psychological therapies for patients with depression. However, the implementation of exercise programs in clinical practice remains challenging. Fatigue, psychomotor retardation, reduced motivation, and decreased physical activity are hallmark symptoms of depression that substantially limit patient adherence to conventional exercise interventions (10). Therefore, developing alternative strategies capable of reproducing the biological benefits of exercise without requiring sustained voluntary effort has become an important therapeutic objective.

Passive exercise modalities, which deliver mechanical stimulation with minimal or no voluntary muscle activity, may provide such an alternative (11). Previous studies have shown that whole-body vibration and other forms of passive mechanical stimulation can improve mood-related behaviors, enhance neuroplasticity, and reduce inflammatory responses in preclinical models (12). Emerging evidence suggests that these antidepressant-like effects may be partially mediated by the gut–brain axis; WBV has been shown to reshape intestinal microbiota composition, enriching short-chain fatty acid-producing taxa such as Alistipes and Lactobacillus, and to enhance anti-inflammatory M2 macrophages and regulatory T cell responses (13, 14). Nevertheless, the underlying mechanisms remain poorly understood, particularly regarding their effects on gut microbiota composition, microbial-associated metabolism, and integrated gut–brain axis signaling. Whether passive mechanical stimulation can induce coordinated neuroendocrine, immune, microbial, and metabolic adaptations comparable to those elicited by active exercise has not been comprehensively investigated.

In the present study, we developed a novel passive exercise system, low-frequency passive vertical oscillation assisted by elastic ropes (LF-PVO-ER), selected for its gentle low-frequency oscillatory stimulation (<30 Hz) that mimics soft-tissue resonance and is well tolerated by subjects with limited physical capacity, thereby potentially optimizing mechanotransduction in the gut wall and associated lymphoid tissues without provoking stress-related cortisol spikes (12, 15). We evaluated its antidepressant-like effects in rats subjected to chronic unpredictable mild stress (CUMS). We further examined its effects on neuroendocrine function, systemic inflammation, hippocampal and colonic histopathology, gut microbiota composition, and fecal metabolomic profiles. By integrating behavioral, biochemical, microbiome, and metabolomic analyses, this study aimed to examine whether LF-PVO-ER is associated with alleviation of depressive-like behaviors and coordinated alterations in gut–brain axis-related markers, and to evaluate its potential as a passive, exercise-mimetic strategy for the management of depression.

## 2 Materials and methods

### 2.1 Animals and experimental design

Six-week-old male Sprague–Dawley rats were housed under standard conditions (12 h light/dark cycle, controlled temperature and humidity) with ad libitum access to food and water. After one-week acclimatization, animals were randomly assigned to six groups: Control (n=6), Control + running wheel (CON-R, n=8), Control + LF-PVO-ER (CON-V, n=7), CUMS (n=6), CUMS + running wheel (CUMS-R, n=6), and CUMS + LF-PVO-ER (CUMS-V, n=6), as described (16). We performed a sensitivity analysis to evaluate the minimum detectable effect size (MDES) under the achieved sample sizes. With n = 6–8 per group (k = 6), α = 0.05, and 80% power, the MDES for one-way ANOVA was Cohen’s f = 0.55–0.65. For pairwise comparisons (two-tailed t-tests), the MDES was Cohen’s d = 1.51–1.80. These thresholds correspond to large effect sizes by conventional criteria (17).

All procedures were approved by the Institutional Animal Ethics Committee (Approval No. 2025IEC016). All experiments were conducted in accordance with the ARRIVE 2.0 guidelines.

### 2.2 Anesthesia and euthanasia

At the end of the experiment, all rats were deeply anesthetized via intraperitoneal injection of sodium pentobarbital (50 mg/kg). The depth of anesthesia was confirmed by the absence of the pedal withdrawal reflex. Under deep anesthesia, blood samples were collected via cardiac puncture. Euthanasia was then performed by gradual-fill CO_2_ inhalation at a displacement rate of 30–70% of the chamber volume per minute, in accordance with the American Veterinary Medical Association (AVMA) guidelines and institutional approval. Death was confirmed by the irreversible cessation of respiration and heartbeat. Immediately thereafter, hippocampal and colonic tissues were harvested for subsequent analyses.

### 2.3 CUMS protocol

Depression-like behavior was induced using a 6-week chronic unpredictable mild stress (CUMS) paradigm including 14 stressors administered in a completely unpredictable daily sequence: 7 h of cage tilting, 5 min of cold swimming (4 °C), 24 h of food or water deprivation, 1 min of tail pinching (1 cm from the tail tip), 12 h on wet bedding, 24 h of single-cage isolation, 5 min of hot water swimming (40 °C), 4 h of physical restraint, 12 h of foreign object placement in the home cage, 5 min of horizontal oscillation, 24 h of bedding deprivation, 12 h of continuous overnight illumination, 24 h of reversed light/dark cycle, and 2 h of white noise exposure (80–90 dB), as previously described (14).

In this 42-day CUMS paradigm, only one stressor was applied per day; no identical stressor was used on consecutive days, and each stressor was repeated no more than 3 times throughout the modeling period to ensure complete unpredictability. Rats in the control groups received no additional stress procedures except for necessary routine husbandry operations. Fecal samples were collected on the final day of CUMS modeling for subsequent analysis.

### 2.4 Exercise interventions

Voluntary running groups had free access to running wheels (6 weeks). LF-PVO-ER groups received passive vertical oscillatory stimulation via elastic rope–assisted apparatus (low-frequency 0.2–1 Hz, low-load <1.5G). Each session consisted of repeated oscillation cycles for 15 min/day over 6 weeks (18).

A custom-designed single-cord passive vertical oscillation apparatus (elastic coefficient k = 16.3 N/m) was employed. The device consisted of an aluminum alloy frame, an elastic cord, and an acrylic restraint basket fitted with a soft harness for adult Sprague-Dawley rats (180–400 g). Two parallel linear guide rails constrained lateral displacement to <0.1 mm. A nylon cord connected the bottom of the restraint basket to a mainspring, which provided a compensatory force to counteract energy dissipation and sustain a stable oscillation frequency. (**Supplementary Figure 1**).

Following body weight measurement, counterweights were adjusted to achieve a total equivalent oscillating mass of approximately 600 g. With the apparatus initially at rest, the basket was manually elevated by 30 cm and then released, initiating passive vertical oscillation of the rat assisted by the elastic cord at a constant natural frequency of (0.2-1Hz).

All rats underwent a 3-day pre-adaptation period (15min/day). The intervention protocol comprised three daily cycles of 5 min oscillation followed by 5 min rest (total 15 min/day), conducted for 6 weeks at a fixed circadian time. Operational parameters followed single-degree-of-freedom spring-mass dynamics. The elastic coefficient was verified prior to each experimental session, and oscillation waveforms were sampled weekly using a triaxial accelerometer (1,000 Hz) to ensure consistency across sessions.

### 2.5 Behavioral tests

Sucrose preference test (SPT), forced swim test (FST), and tail suspension test (TST) were conducted to assess anhedonia and behavioral despair (19). All three behavioral tests were conducted 24 h post the 6-week intervention, with a 24 h interval between each test to avoid cross-stress interference, and performed in a group-stratified random order by investigators blinded to animal group allocation.

#### 2.5.1 Sucrose preference test (SPT)

Baseline measurements were obtained during a 7-day acclimation period, wherein rats were housed individually and presented with two identical bottles for 12 h: one containing a 1% (w/v) sucrose solution and the other containing plain tap water. Prior to the preference test, rats were deprived of food and water for a period of 12 hours. During the test, rats were individually housed and provided with simultaneous access to two pre-weighed bottles: one containing 200 mL of 1% sucrose solution and the other containing 200 mL of tap water, for a duration of 12 hours. The positions of the two bottles were switched after 6 hours to control for potential side preferences. The consumption of both sucrose solution and tap water was determined by measuring the change in weight of each bottle. Sucrose preference (SP) was calculated using the following formula: SP (%)= [Sucrose intake (g)/(Sucrose intake (g) + Water intake (g))] × 100%.

#### 2.5.2 Forced swim test (FST)

Rats were individually placed in one plexiglass cylinder (diameter 40 cm, height 60 cm) filled with 40 cm deep water (23–25 °C) for 6 min and videotaped in every session using the BAS-100 Behavioral Analysis Software (TECHMAN, China). Immobility was defined as the cessation of all active swimming and struggling movements, with the rat only making movements essential to keep its head above the water surface.

#### 2.5.3 Tail suspension test (TST)

Each rat was suspended by taping the tail 1–2 cm from its tip, positioning the head downward with the nose approximately 10 cm above the laboratory bench surface, for a test duration of 6 minutes. Immobility was defined as a complete lack of active, escape-directed movements, with the animal remaining motionless except for necessary respiratory motions. The total duration of immobility during the 6-minute test was automatically quantified using the BAS-100 Behavioral Analysis System (TECHMAN, China) to assess depressive-like behavioral despair.

### 2.6 Biochemical assays

Serum 5-HT, BDNF, corticosterone, IL-6, TNF, and LPS were measured using ELISA kits according to manufacturer protocol (20).

### 2.7 Histological analysis

Hippocampal and colonic tissues were fixed, sectioned, and stained with hematoxylin and eosin (H&E) for morphological evaluation. Histological changes were quantified using a semi-quantitative scoring system by two observers blinded to the experimental groups; all sections were coded by an independent investigator prior to assessment. Scoring was performed on randomly selected high-power fields.

Hippocampal damage was evaluated on H&E-stained slides using a semiquantitative scoring system modified from (21): score 0 = no obvious damage; score 1 = loss of single neurons, involving <10% of neurons; score 2 = one or more segmental areas with neuronal loss involving <20% of neurons; score 3 = one or more segmental areas with neuronal loss involving 20 - 50% of neurons; score 4 = one or more segmental areas with neuronal loss involving >50% of neurons.

Colonic injury was assessed by scoring two parameters—mucosal epithelial damage and inflammatory cell infiltration—each on a 0–4 scale (**Table 1**). The total histopathological score (range: 0–8) was calculated as the sum of the two sub-scores (22).

**Table 1:** Histologic scoring criteria for colonic injury.

Score	Epithelial lesion score degree of epithelial damage	Infiltration lesion score degree of infiltration
0	normal	none
1	loss of goblet cells	infiltration surrounding crypt basis
2	loss of goblet cells in large areas	infiltration into the mucosa
3	loss of crypts	widespread infiltration into the mucosa
4	loss of crypts in large areas	infiltration into the submucosa

### 2.8 Microbiota analysis

Fecal total DNA was extracted using Tiangen TGuide S96 kit. 16S rRNA V3-V4 region (338F/806R barcoded primers) was sequenced on Illumina NovaSeq 6000 (2×250 bp), with minimum valid sequencing depth ≥ 50,000 reads. Raw data was processed via QIIME2–2023 pipeline: DADA2 denoising, chimera removal, ASV generation, and taxonomic annotation against SILVA 138 database. After quality control, the average sequence retention rate was 82.3%, the high-quality sequence length of all samples was concentrated in 400~430 bp, and a total of 4102 valid ASVs were finally obtained. Rarefaction curves were plotted based on ASV abundance, and the curves of all samples reached the plateau stage when the sequencing volume reached 30000 reads, confirming that the current sequencing depth fully covered most bacterial species in the samples.

Alpha diversity (Shannon, Simpson, Pielou’s evenness, Chao1) and beta diversity (PCA) were analyzed on Novogene NovoMagic platform. Differential taxa were preliminarily screened by Kruskal–Wallis test, and the original p-values were corrected for multiple testing using the Benjamini-Hochberg false discovery rate method, with adjusted *p* < 0.05 defined as statistical significance. LEfSe was used for biomarker screening with unified criteria: LDA score = 3, adjusted *p* < 0.05.

### 2.9 Metabolomics

Fecal metabolites were extracted with ice-cold 80% methanol. Pooled QC samples were injected every 6 runs to monitor system stability. LC-MS/MS was performed on Thermo UHPLC-Orbitrap Exploris 480 system using a Waters HSS-T3 column (2.1×100 mm, 1.8 μm) for gradient elution. Metabolites were annotated against mzCloud and HMDB databases.

All inter-group OPLS-DA models were Pareto-scaled, with strictly limited principal components to avoid artificial overfitting. A 200-iteration permutation test verified that all models reached permutation p < 0.05, with R²Y > 0.92 and Q² > 0.75. Raw two-tailed t-test p-values were corrected for multiple testing via the Benjamini-Hochberg false discovery rate method. Differential metabolites were screened by combined criteria: OPLS-DA VIP > 1, adjusted p < 0.05, FC > 1.2 or < 0.83. KEGG pathway enrichment was conducted for differential metabolites (23).

### 2.10 Statistical analysis

All statistical analyses were conducted in GraphPad Prism 9.0 and NovoMagic platform. Normality (Shapiro–Wilk) and variance homogeneity (Brown–Forsythe) were tested beforehand. For behavioral and biochemical data with a 2×2 factorial design, two-way ANOVA was used to examine the main effects of stress, intervention, and their interaction. For repeated body weight measurements, repeated-measures ANOVA was applied. Parametric data was analyzed by one-way ANOVA with appropriate *post-hoc* tests; non-parametric data was processed via Kruskal–Wallis test. Spearman correlation was used for microbiota-metabolite association analysis. Data are presented as mean ± SEM, and p < 0.05 was considered statistically significant (24).

## 3 Results

### 3.1 LF-PVO-ER improves depressive-like behaviors

CUMS induced significant reductions in body weight gain and sucrose preference, along with increased immobility time in both the forced swim test (FST) and tail suspension test (TST) (p < 0.05). Both LF-PVO-ER and voluntary running significantly reversed these behavioral changes (**Figures 1B–E**). Effect size analysis revealed comparable effects on body weight and FST immobility (Cohen’s d ≈ 0), but notable differences in sucrose preference (d = -0.87, 95% CI [-1.89, 0.18]) and TST immobility (d = 2.41, 95% CI [0.98, 3.77]). The experimental workflow is summarized in **Figure 1A**.

**Figure 1:**
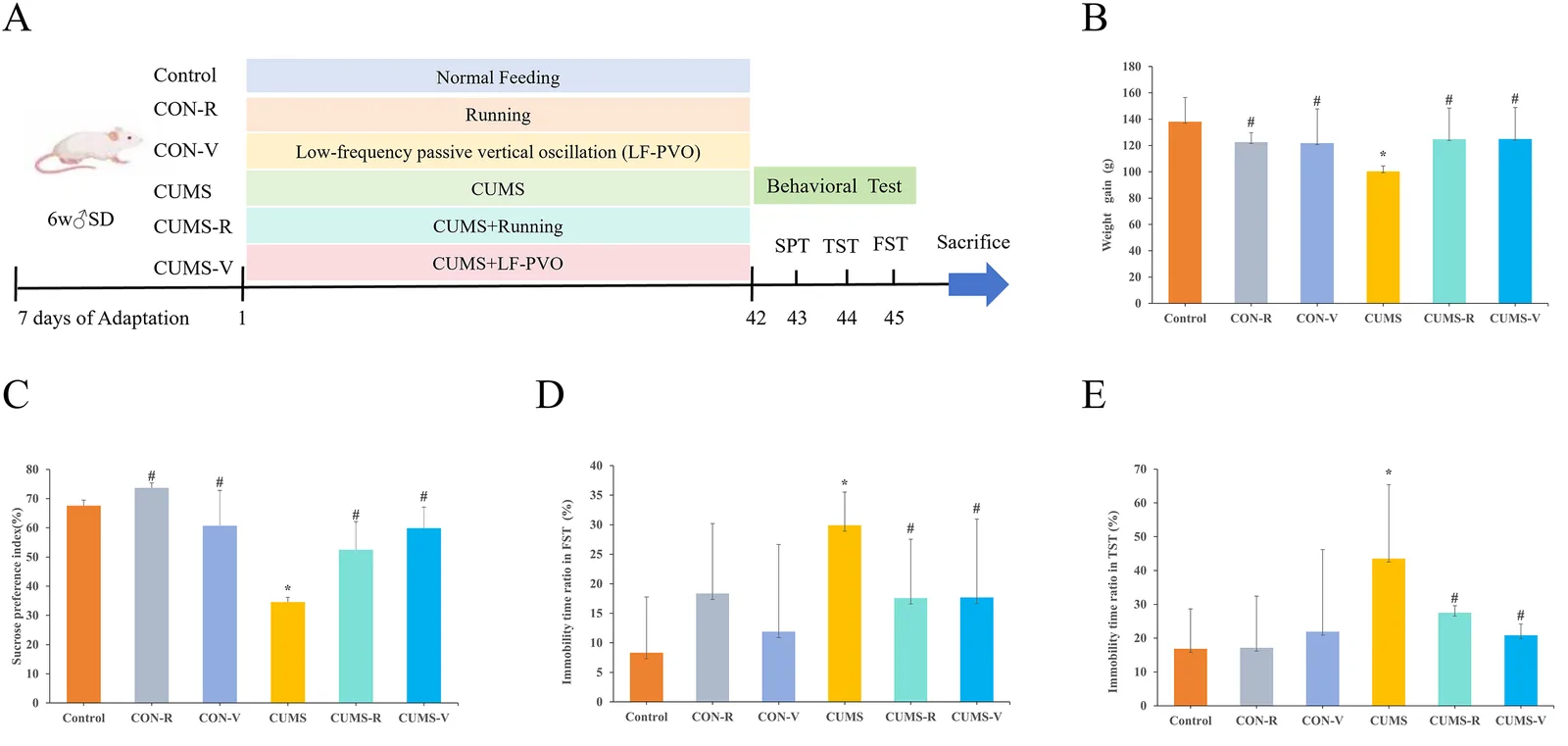
LF-PVO-ER ameliorates depressive-like behaviors in CUMS rats. **(A)** Experimental design schematic. **(B)** Body weight changes during the 6-week experimental period. **(C)** Sucrose preference percentage in the Sucrose Preference Test (SPT). **(D)** Immobility time in the Forced Swim Test (FST). **(E)** Immobility time in the Tail Suspension Test (TST). *p < 0.05 vs. Control group; *^#^p < 0.05* vs. CUMS group.

### 3.2 LF-PVO-ER normalizes neuroendocrine and inflammatory markers

CUMS decreased serum 5-HT and BDNF and increased corticosterone levels (p < 0.05). It also elevated IL-6, TNF, and LPS levels.

LF-PVO-ER significantly reversed all these alterations compared with the CUMS group (p < 0.05) (**Figures 2A–F**), indicating restoration of neuroendocrine balance and suppression of systemic inflammation. No significant differences were observed between CON, CON-R, and CON-V groups.

**Figure 2:**
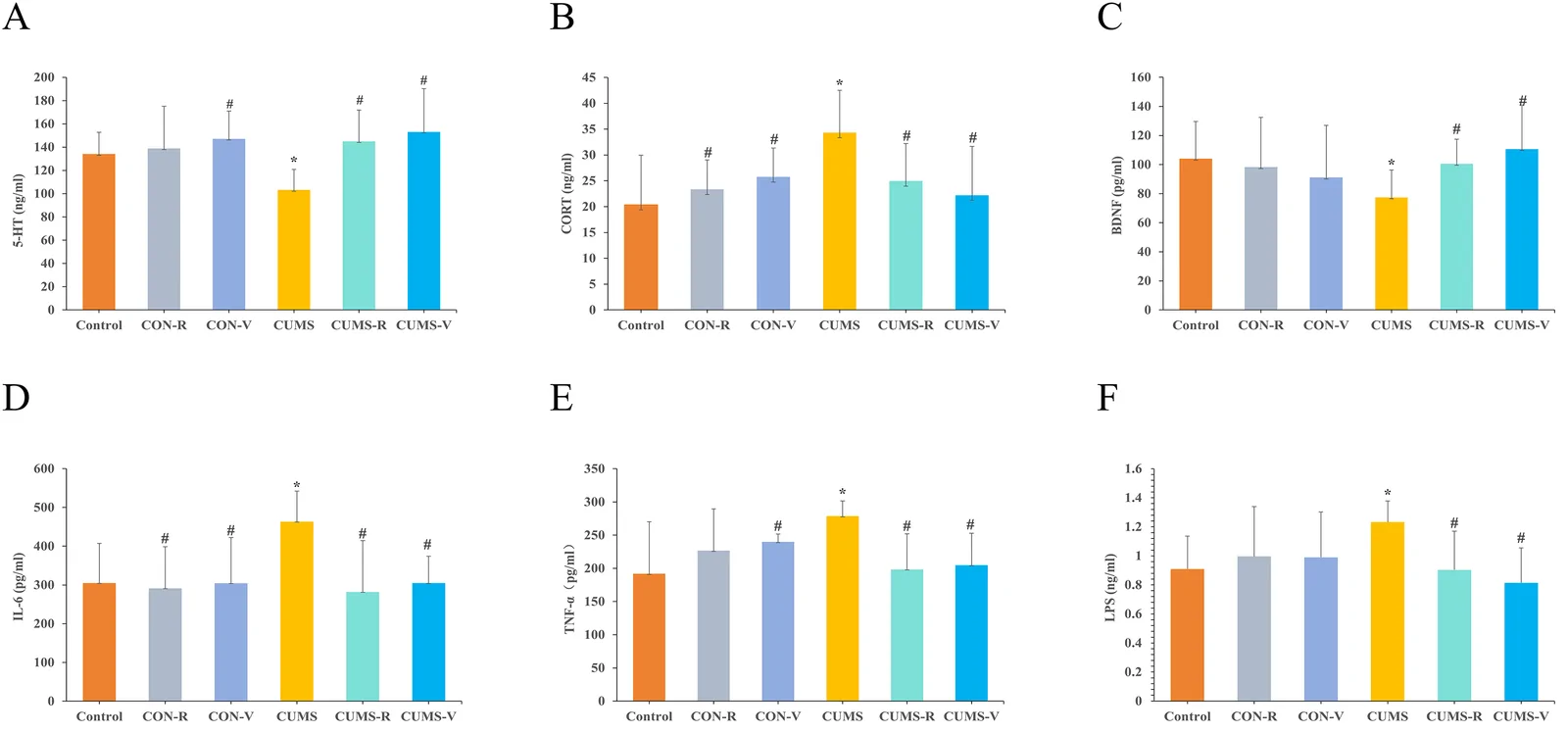
The levels of neurotransmitters and inflammation factors. **(A)** 5-HT. **(B)** CORT. **(C)** BDNF. **(D)** IL-6. **(E)**TNF-α. **(F)** LPS. *p < 0.05 vs. Control group; *^#^p < 0.05* vs. CUMS group.

### 3.3 LF-PVO-ER attenuates hippocampal and colonic injury

Histopathological analysis revealed that CUMS induced marked hippocampal neuronal disorganization and colonic mucosal damage.

LF-PVO-ER and voluntary running partially restored hippocampal structure and improved colonic mucosal integrity (**Figures 3A, B**), indicating protective effects on both central and peripheral tissues.

**Figure 3:**
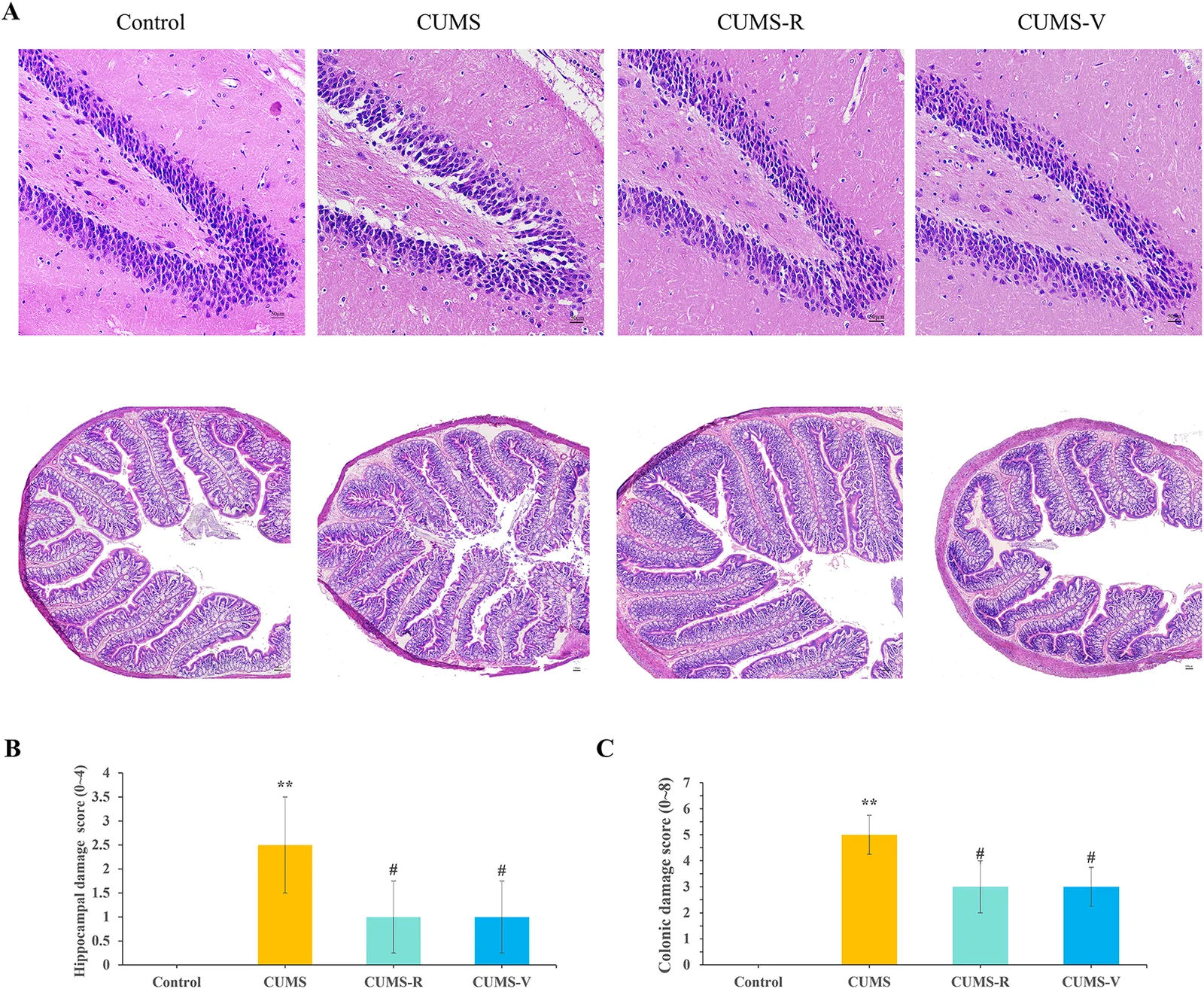
Histopathological damage scores in the hippocampus and colon. **(A)** H&E-stained sections of the hippocampus (scale bar = 50 μm); H&E-stained sections of the colon (scale bar = 100 μm). **(B)** Hippocampal histopathological damage score. **(C)** Colonic histopathological damage score. **(B, C)** Data are presented as median with interquartile range (IQR), (*n* = 6 per group). Statistical analysis was performed using the Kruskal-Wallis H test followed by Dunn’s *post-hoc* test with Bonferroni correction. For the hippocampus, the histopathological score differed significantly among the four groups (*H* = 20.35, *p* = 0.0001). CUMS exposure significantly increased hippocampal damage compared to the Control group (*p* < 0.0001). Both CUMS-R and CUMS-V treatments significantly attenuated hippocampal injury compared to the CUMS group (CUMS-R: *p* = 0.0168; CUMS-V: *p* = 0.0168). For the colon, the histopathological score differed significantly among the four groups (*H* = 21.07, *p* = 0.0001). CUMS exposure significantly increased colonic damage compared to the Control group (*p* < 0.0001). Both CUMS-R and CUMS-V treatments significantly attenuated colonic injury compared to the CUMS group (CUMS-R: *p* = 0.0207; CUMS-V: *p* = 0.0011). **p < 0.001 vs. Control group; *^#^p < 0.05 vs.* CUMS group.

### 3.4 LF-PVO-ER restores gut microbiota dysbiosis

CUMS significantly reduced microbial diversity and altered community structure.

Alpha diversity indices (Shannon, Simpson, Pielou’s evenness, Chao1) showed significant impairment in the CUMS group, which was partially reversed by LF-PVO-ER (**Figures 4A–E**). Beta diversity analysis (PCA) demonstrated clear separation among groups, indicating distinct microbial community structures (**Figure 4F**). Venn diagram analysis further highlighted differences in shared and unique genera among groups (**Figure 4G**). At the phylum level, CUMS increased the Firmicutes-to-Bacteroidota ratio, which was partially normalized by LF-PVO-ER (**Figure 4I**). At the genus level, LF-PVO-ER increased SCFA-associated taxa (e.g., Lachnospiraceae_NK4A136_group, Romboutsia, Monoglobus) and reduced stress-associated taxa such as Enterococcus and Ligilactobacillus (**Figure 4J**). LEfSe analysis identified distinct microbial biomarkers among groups (LDA score > 3), highlighting taxa significantly enriched in CUMS and LF-PVO-ER conditions (**Figures 4K, L**).

**Figure 4:**
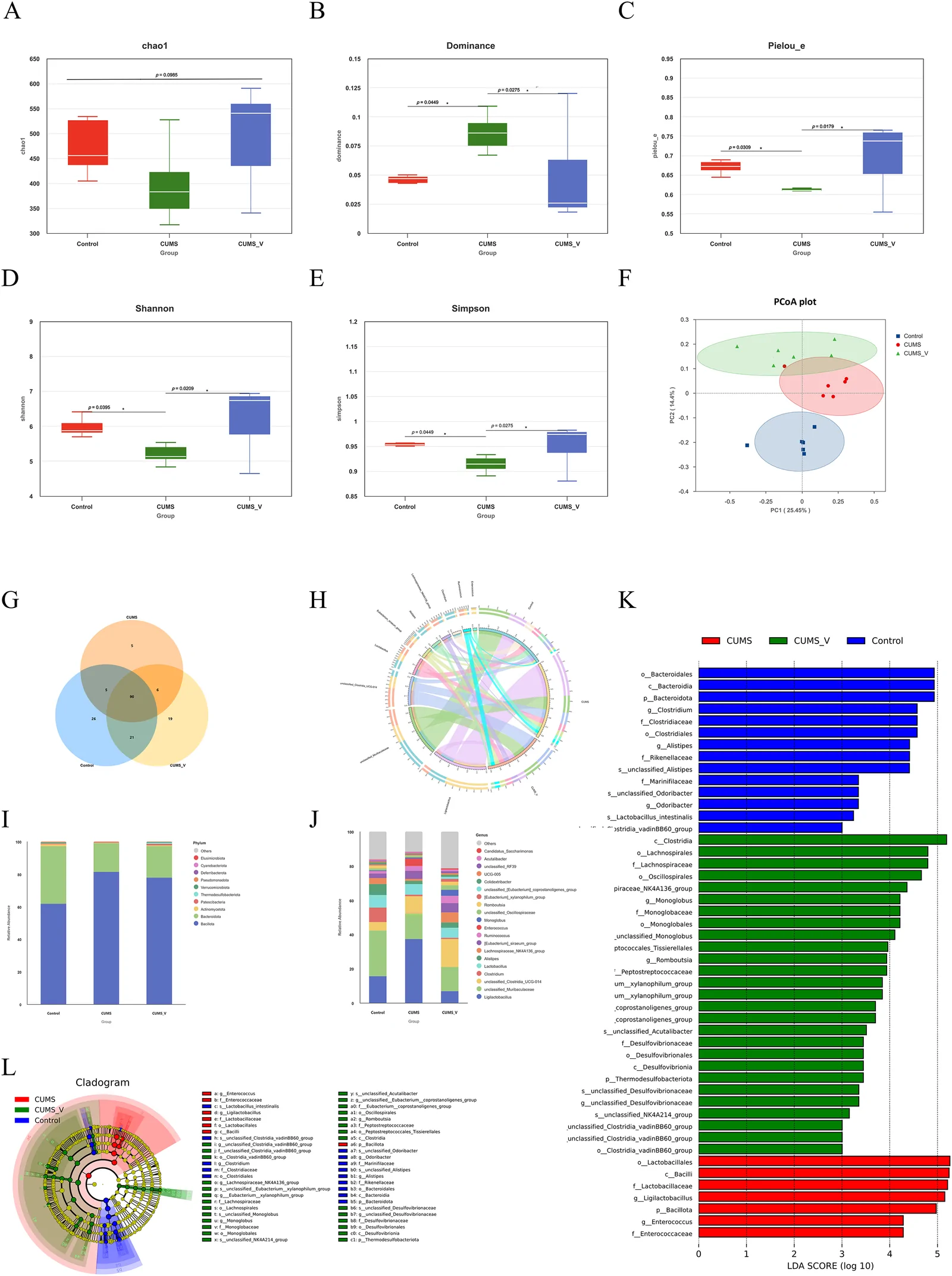
Gut microbial characteristics of Control, CUMS, and CUMS-V. **(A)** Chao1 index. **(B)** Dominance index. **(C)** Pielou_e index. **(D)** Shannon index. **(E)** Simpson index. **(F)** Principal component analysis (PCA) showed that the gut microbiota composition of CUMS-induced depressive-like rats was markedly distinct from that of the Control group, and LF-PVO-ER treatment partially restored the microbial composition. **(G)** Venn diagram for taxonomy of gut microbes based on genus level. **(H)** Circus circle diagram of the top 10 relative abundances in intestinal microbial classification based on genus level. **(I)** The gut microbiota compositions among the experimental groups at the phylum level. **(J)** The relative abundance of gut microbes at the genus level. **(K)** Histogram of LDA scores identifying significantly enriched taxa (LDA score > 3.0) in each group. **(L)** Evolutionary Cladogram from LEfSe Analysis. **(A–E)** Significant differences among the three groups were determined via Kruskal-Wallis test. *p < 0.05 vs. CUMS group (*n* = 6 per group).

Taxonomic composition profiling further supported these shifts (**Supplementary Figure 2**).

### 3.5 LF-PVO-ER modulates fecal metabolomic profiles

PLS-DA and OPLS-DA analyses demonstrated clear separation between groups, indicating distinct metabolic phenotypes (**Figures 5A–E**). A total of 87 differential metabolites were identified between CUMS and Control, and 53 between CUMS-V and CUMS (VIP > 1.0, p < 0.05) (**Figures 5G, H**; **Supplementary Tables S1**, **S2**). Key metabolites included alterations in amino acid derivatives, lipid-related compounds, bile acid metabolites, and antioxidant-related molecules. KEGG pathway enrichment analysis revealed that CUMS primarily affected amino acid and energy metabolism pathways (**Figure 5I**), whereas LF-PVO-ER regulated ubiquinone biosynthesis and α-linolenic acid metabolism (**Figure 5J**). The repeatability of core differential metabolites was validated via technical replicates.

**Figure 5:**
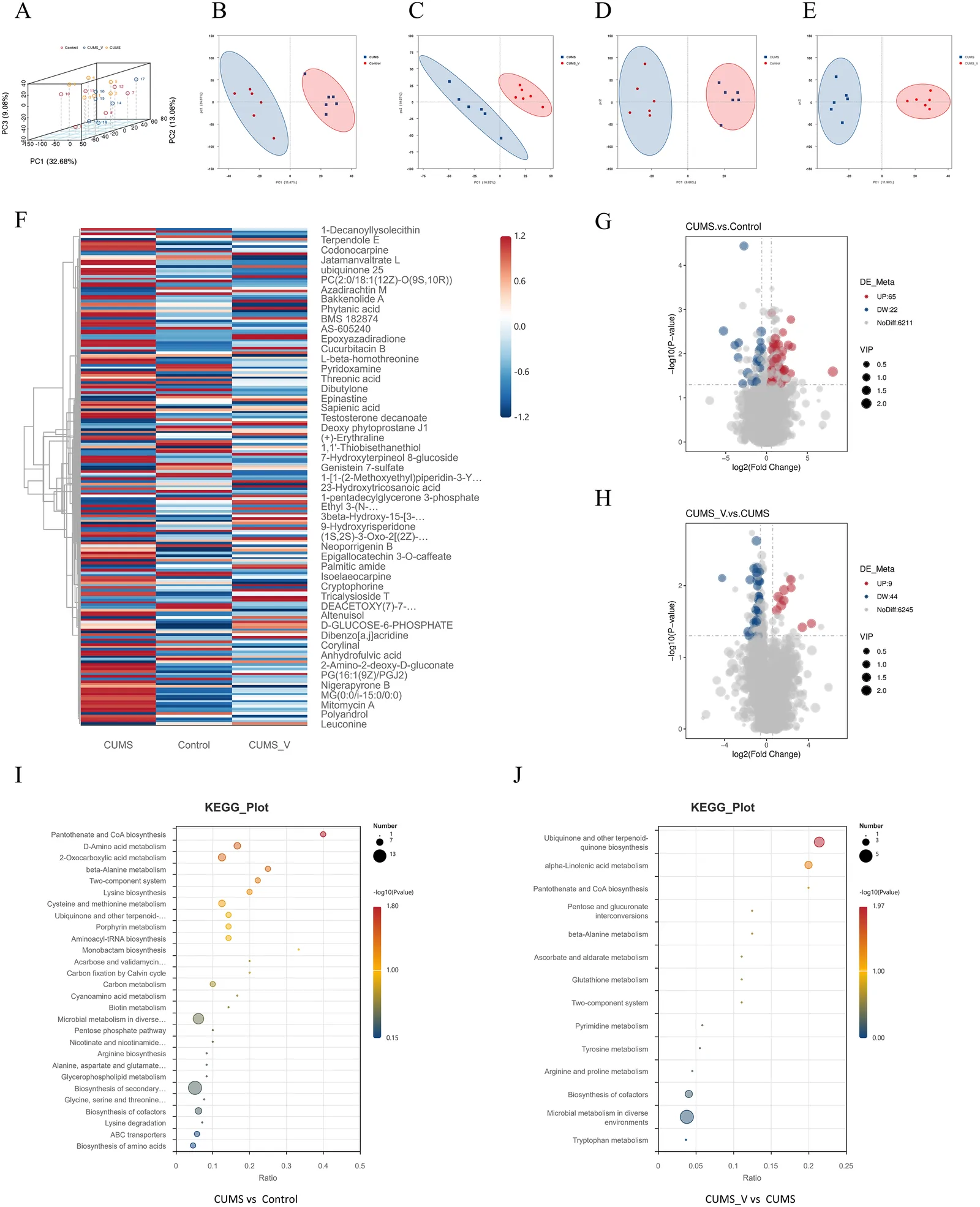
Multivariate analysis of metabolic profiles in the Control, CUMS, and CUMS-V groups. **(A–C)** The evaluation scatter plots of different groups from the PLS-DA data. **(D)** Control and CUMS pairwise comparison, OPLS-DA evaluation scatter plots. **(E)** CUMS and CUMS-V pairwise comparison, OPLS-DA evaluation scatter plots. **(F)** Heatmaps showing the relative abundance of significantly differential metabolites identified from OPLS-DA comparisons. Differential Metabolite Volcano Plot: **(G)** Control *vs*. CUMS; **(H)** CUMS-V *vs*. CUMS. KEGG pathway enrichment analysis for the differential metabolites identified in the corresponding comparisons (*n* = 6 per group): **(I)** CUMS *vs*. Control; **(J)** CUMS-V *vs*. CUMS.

### 3.6 Integrated microbiota–metabolite–host associations

Spearman correlation analysis demonstrated significant associations among gut microbiota, fecal metabolites, and neuroimmune markers (**Figures 6**, **7**). SCFA-producing genera were positively associated with 5-HT and BDNF and negatively associated with IL-6, TNF, and LPS. In contrast, stress-associated taxa showed opposite correlations. Similarly, several metabolites were significantly correlated with neuroendocrine and inflammatory indices, supporting coordinated gut–brain–immune interactions (**Figures 7**, **8**). These findings are correlative and do not establish causality.

**Figure 6:**
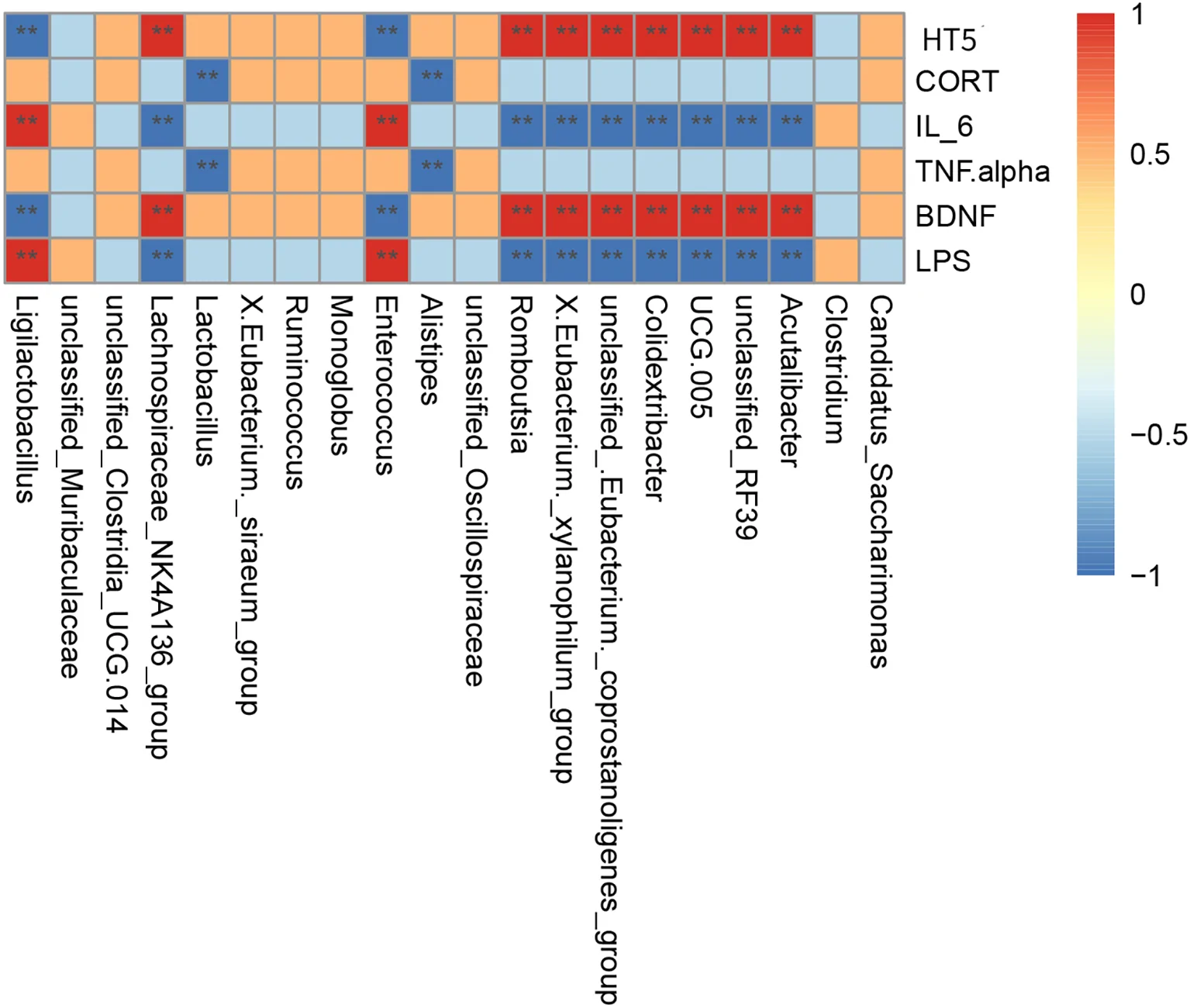
Associations of gut microbes with neurotransmitters and inflammation factors based on Spearman correlation analysis. Visualization of Spearman’s rank correlation in the form of a heatmap of 20 genera and neurotransmitters, with inflammation in the colon. *p < 0.05, **p < 0.01.

**Figure 7:**
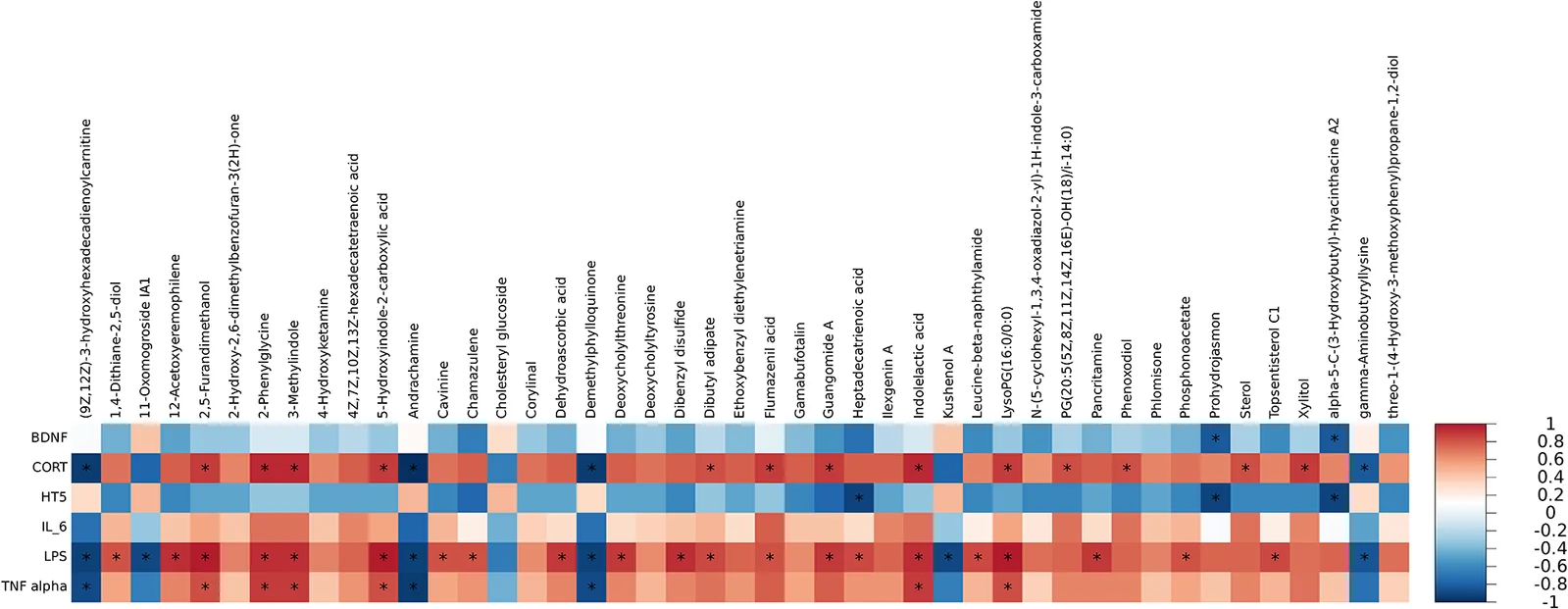
Associations of gut metabolites with neurotransmitters and inflammation factors based on Spearman correlation analysis. *p < 0.05.

**Figure 8:**
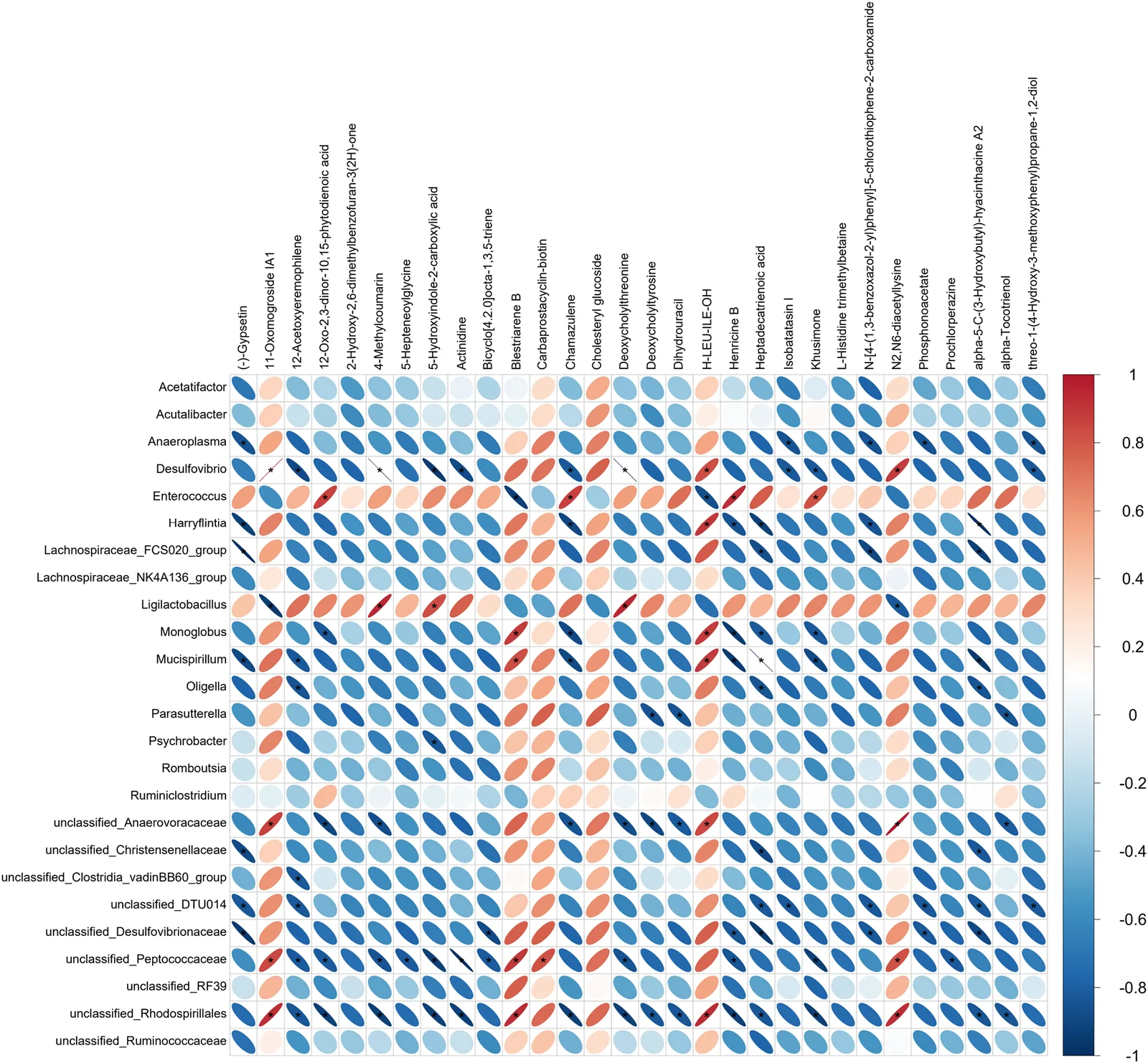
Associations of gut based on Lefse-genus and metabolites based on OPLS-DA. *p < 0.05.

## 4 Discussion

The present study demonstrated that LF-PVO-ER, a novel passive mechanical stimulation modality, was associated with alleviation of depressive-like behaviors in a chronic unpredictable mild stress (CUMS) rat model. Improvements in anhedonia and behavioral despair were accompanied by changes in neuroendocrine markers consistent with restored homeostasis, attenuation of systemic inflammation, preservation of hippocampal and colonic morphology, remodeling of gut microbiota composition, and alterations in microbial-associated metabolic pathways. Importantly, these beneficial effects were broadly comparable to those observed with voluntary wheel running, suggesting that passive mechanical stimulation may reproduce several biological effects traditionally attributed to active exercise.

Exercise has long been recognized as an effective non-pharmacological intervention for depression (24); however, its clinical implementation is often limited by reduced motivation, fatigue, and physical inactivity, which are core symptoms of depressive disorders. Consequently, passive exercise strategies that require minimal voluntary effort have attracted increasing attention. Previous studies have reported that whole-body vibration and other forms of passive mechanical stimulation improve mood-related behaviors and cognitive function, although the underlying mechanisms remain poorly understood (25). Our findings extend the concept by demonstrating that low-frequency passive vertical oscillation not only improves behavioral outcomes but also simultaneously modulates neuroendocrine, immune, microbial, and metabolic pathways. These observations suggest that passive mechanical stimulation may warrant further preclinical investigation as a potential alternative for individuals unable to engage in conventional exercise because of severe depression, physical disability, or chronic illness.

One of the principal findings is the alteration of peripheral neuroendocrine markers following LF-PVO-ER intervention. Chronic stress significantly reduced circulating serotonin and BDNF while increasing corticosterone concentrations (26), whereas LF-PVO-ER largely reversed these alterations. we acknowledge that serum serotonin primarily reflects peripheral sources such as enterochromaffin cells and platelets, and that serum BDNF does not necessarily mirror central BDNF concentrations (27, 28). Consequently, these peripheral changes cannot be directly interpreted as evidence of central neuroplasticity. Nevertheless, they may reflect broader systemic adaptations that could indirectly influence brain function, and their concurrent modulation by LF-PVO-ER warrants further investigation in relation to central serotonergic and neurotrophic signaling.

The reduction in corticosterone is further consistent with normalization of hypothalamic–pituitary–adrenal (HPA) axis activity. Chronic activation of the HPA axis contributes not only to emotional disturbances but also to neuroinflammation, impaired hippocampal plasticity, and increased intestinal permeability (29). Consequently, restoration of glucocorticoid homeostasis may represent an upstream mechanism associated with many of the beneficial changes observed in this study. Although the precise signaling pathways remain to be determined, mechanical stimulation may influence autonomic nervous system activity and neuroendocrine regulation, thereby reducing chronic stress responses and promoting systemic homeostasis.

Increasing evidence indicates that depression is accompanied by chronic low-grade inflammation, characterized by elevated circulating pro-inflammatory cytokines such as IL-6 and TNF. These cytokines can influence neurotransmitter metabolism, impair synaptic plasticity, and activate microglia, thereby contributing to depressive symptom development (30). Moreover, elevated serum hypersensitive C-reactive protein has been independently associated with cognitive dysfunction in first-episode, drug-naïve patients with major depressive disorder (31), suggesting that inflammatory activation may exert concurrent detrimental effects on both mood and cognitive domains. In the present study, LF-PVO-ER significantly reduced circulating IL-6 and TNF concentrations, suggesting suppression of systemic inflammatory activation. Interestingly, serum LPS was also markedly reduced following intervention. Elevated circulating LPS reflects impaired intestinal barrier integrity and translocation of bacterial endotoxins into the circulation, which subsequently amplifies inflammatory responses through Toll-like receptor-mediated signaling (32). Together with the observed improvement in colonic histology, these findings suggest that LF-PVO-ER may strengthen intestinal barrier function, which is associated with reduced systemic endotoxemia and may interrupt the positive feedback loop between gut dysfunction, inflammation, and depressive pathology.

Histological analyses further support the protective effects of LF-PVO-ER on both central and peripheral tissues. Chronic stress produced characteristic neuronal disorganization within the hippocampus together with disruption of colonic mucosal architecture, whereas both abnormalities were substantially alleviated following intervention. The hippocampus is particularly vulnerable to chronic glucocorticoid exposure and inflammatory injury and plays a critical role in emotional regulation and stress adaptation (33). Meanwhile, preservation of intestinal structure is consistent with reduced gut permeability and improved mucosal integrity. These observations reinforce the concept that depression involves simultaneous dysfunction of the central nervous system and gastrointestinal tract, highlighting the importance of considering depression as a systemic disorder associated with bidirectional gut–brain communication.

An important strength of this study is the demonstration that LF-PVO-ER partially restores stress-induced gut microbial dysbiosis. Chronic stress reduced microbial diversity and altered overall community composition, whereas passive mechanical stimulation increased microbial richness and shifted the microbiota towards taxa generally associated with intestinal health (34). In particular, enrichment of Lachnospiraceae_NK4A136_group, Romboutsia, and Monoglobus is noteworthy because many members of these genera are putative short-chain fatty acid (SCFA) producers (35). However, as fecal SCFA concentrations were not directly quantified in this study, any inference regarding butyrate production or SCFA-mediated mechanisms remains speculative. In future studies to confirm SCFA output, these metabolites might contribute to epithelial barrier maintenance and immune regulation (8); at present, however, the observed microbial shifts can only be considered as putative indicators of altered fermentation capacity. Consistent with this, recent clinical metagenomic evidence in drug-naïve patients with major depressive disorder has identified *Bacteroides cellulosilyticus* as a potentially protective species whose depletion correlates with suicidal ideation severity, possibly mediated through enhanced carbohydrate transport and SCFA production (36). Conversely, reductions in stress-associated taxa, including Enterococcus and Ligilactobacillus, suggest partial restoration of microbial homeostasis. Although the functional roles of individual bacterial genera remain context-dependent, the overall microbial changes observed here are consistent with an improved intestinal ecosystem that may contribute to the behavioral benefits of LF-PVO-ER.

The metabolomic analysis complements these microbial findings by demonstrating coordinated alterations in host metabolic pathways. Depression has consistently been associated with disturbances in amino acid metabolism, lipid metabolism, mitochondrial function, and oxidative stress (37). Cross-diagnostic metagenomic studies have further revealed that multi-kingdom microbial communities—including bacteria, fungi, archaea, and viruses—exhibit coordinated functional disturbances, such as increased tryptophan metabolism and decreased amino acid biosynthesis, which are intimately associated with clinical symptom severity and cognitive function in psychiatric disorders (38). LF-PVO-ER influenced several metabolic pathways, including amino acid metabolism, ubiquinone biosynthesis, α-linolenic acid metabolism, and bile acid metabolism. Alterations in amino acid metabolism are particularly relevant because tryptophan serves as the precursor for serotonin synthesis and is diverted towards the kynurenine pathway during chronic inflammation (39, 40). However, given the correlative nature of metabolomic data and the lack of direct central neurotransmitter measurements, any link between these peripheral metabolic changes and serotonergic signaling remains speculative. Likewise, changes in lipid-related pathways and ubiquinone biosynthesis may reflect improved mitochondrial function and reduced oxidative stress, although these interpretations are speculative given the absence of direct functional assays (41). Although the biological significance of individual metabolites requires further investigation, the overall metabolic profile supports extensive host–microbiota co-metabolic remodeling following passive mechanical stimulation.

The integrated correlation analyses provide additional support for coordinated gut–brain interactions. Putative SCFA-associated bacterial genera were positively correlated with serum serotonin and BDNF while showing negative associations with inflammatory cytokines and LPS (42). Conversely, stress-associated taxa displayed opposite relationships. Similar associations were identified between differential metabolites and neuroimmune markers. These findings collectively suggest that the antidepressant-like effects of LF-PVO-ER are associated with the restoration of gut microbial and metabolic homeostasis, which may be a component of the underlying mechanism. Although these analyses cannot establish causal relationships, they support the hypothesis that LF-PVO-ER is associated with coordinated changes across microbial, metabolic, endocrine, and immune networks. Future studies employing causal approaches, including fecal microbiota transplantation, antibiotic depletion, germ-free animals, or targeted metabolite supplementation, will be necessary to determine whether microbial remodeling directly mediates the antidepressant effects of LF-PVO-ER.

Several limitations should be acknowledged. First, only male rats were included, and sex-specific responses cannot be excluded given the recognized influence of sex hormones on depression and gut microbiota composition. Second, the study relied on a single CUMS model, and validation in additional depression models would strengthen the generalizability of the findings. Third, the multi-omics analyses were primarily correlative and therefore cannot establish mechanistic causality between microbial alterations and behavioral improvements. Fourth, spontaneous locomotor activity (e.g., open field test) and anxiety-related or cognitive behavioral assessments were not conducted. Without these measures, we cannot exclude potential confounding effects of altered general locomotion, anxiety states, or cognitive function on the observed behavioral outcomes; consequently, the specificity of LF-PVO-ER’s antidepressant-like effects remains to be clarified. Finally, although LF-PVO-ER showed antidepressant-like effects similar to those observed with voluntary exercise in this preclinical model, clinical studies are required to determine its feasibility, safety, and therapeutic efficacy in patients with depression.

The present study employed n = 6–8 animals per group. A sensitivity analysis indicated that this sample size provided 80% power to detect large effect sizes (Cohen’s f ≈ 0.55–0.60) for behavioral endpoints. This is adequate for CUMS-induced depression models, where behavioral deficits (e.g., reduced sucrose preference) typically exhibit large effect sizes (f = 0.60–1.20) (43, 44). However, for microbiome and metabolomic endpoints, effect sizes may be smaller and more variable due to high inter-individual biological variation (45). Consequently, the study may have been underpowered to detect modest differences in microbial alpha-diversity indices or low-abundance metabolites, increasing the risk of type II errors (false negatives). Significant findings in these omics endpoints should be interpreted as robust large effects, whereas non-significant trends may warrant validation in larger cohorts. We have reported exact p-values and effect sizes (partial η² or Cohen’s d) to allow readers to assess the magnitude of observed effects independent of sample size (46).

In conclusion, the current study demonstrated that LF-PVO-ER was associated with robust antidepressant-like effects and coordinated alterations in neuroendocrine markers, systemic inflammation, intestinal integrity, gut microbiota composition, and microbial-associated metabolism in a rodent model. Rather than being limited to a single pathway, these findings suggest that passive mechanical stimulation is associated with changes across multiple interconnected components of the gut–brain axis. Although these preclinical findings are encouraging, LF-PVO-ER is premature to recommend for clinical use until its chronic safety and feasibility have been established in human studies. Further mechanistic and clinical investigations are warranted to clarify the biological pathways involved and to evaluate its translational potential for the treatment of depression.

## Data Availability

The data presented in the study are deposited in the NCBI repository, BioProject accession number PRJNA1538061 (https://www.ncbi.nlm.nih.gov/bioproject/1538061). The corresponding BioSample accession numbers are SAMN63808692 -SAMN63808709.
