# Supplementary material for: Low-frequency passive mechanical stimulation produces antidepressant-like effects in rats involving gut–brain axis modulation
Source: Front Immunol. 2026 Oct 12;17:1928978. doi: 10.3389/fimmu.2026.1928978 (PMC13696317; doi:10.3389/fimmu.2026.1928978)
Supplement: Supplementary file 1 [file DataSheet1.docx]

Supplementary Material

# 1 Supplementary Figures and Tables

## 1.1 Supplementary Figures

**Supplemental Figure 1.** Schematic of the elastic rope-assisted low-frequency vertical oscillation apparatus for rats. This unpowered mainspring-driven device operates at 0.2–1 Hz with a low load < 1.5 G, and is suitable for 180–400 g rats to receive stable long-term vertical mechanical stimulation.

**Supplemental Figure 2.** LF-PVO-ER intervention regulated the gut microbiota in CUMS-induced depressive-like rats.

**(A)** Heatmap of the gut microbiome in genus level. **(B)** Heatmap of abundance distribution between groups in genus level. **(C)** The comparison of the relative abundance of gut microbial community members was conducted at the genus level among three groups. Data are expressed as mean±S.E.M. (*n*= 6 per group). *# p* < 0.05 vs. CUMS group; ** p* < 0.05 vs. Control group

# 1.2 Supplementary Tables

Supplement materials

Table S1 The differential metabolites between the Control and CUMS group

| Name | Formula | MolecularWeight | m/z | RT (min) | Pvalue | VIP | Up.Down |
| --- | --- | --- | --- | --- | --- | --- | --- |
| Sterol | C17H28O | 248.21401 | 247.2064 | 7.66 | 3.65E-05 | 1.406079 | down |
| Kushenol I | C26H30O7 | 454.19916 | 435.181 | 5.507 | 0.001201 | 1.137693 | up |
| Cyprofuram | C14H14ClNO3 | 279.06622 | 278.0606 | 4.879 | 0.001689 | 1.201485 | up |
| Gly Arg Trp | C19H27N7O4 | 417.21245 | 418.2181 | 5.082 | 0.003056 | 1.568446 | down |
| Aspartyllysine | C10H19N3O5 | 261.13247 | 244.1291 | 1.961 | 0.003167 | 1.85156 | down |
| ISOPROPYL BETA-D-GLUCOPYRANOSIDE | C9H18O6 | 222.11034 | 221.1026 | 4.954 | 0.003299 | 1.106374 | up |
| Arterolane | C22H36N2O4 | 392.26751 | 393.2749 | 6.443 | 0.004537 | 1.605225 | up |
| (3E,5E)-6,10-dimethylundeca-3,5,9-trien-2-one | C13H20O | 192.15141 | 175.1482 | 8.439 | 0.005338 | 1.039663 | down |
| H-LEU-ILE-OH | C12H24N2O3 | 244.17869 | 245.186 | 2.122 | 0.00564 | 1.294966 | down |
| Deacetylbowdensine | C17H21NO5 | 319.14197 | 318.1371 | 5.922 | 0.005906 | 1.214931 | up |
| Epigallocatechin 3-O-caffeate | C24H20O10 | 468.10565 | 467.1017 | 5.362 | 0.006089 | 1.298507 | up |
| 3-(E)-Coumaroylbetulinaldehyde | C39H54O4 | 586.40221 | 585.4006 | 7.005 | 0.006131 | 1.308404 | up |
| Alnespirone | C26H38N2O4 | 442.28316 | 443.2881 | 7.104 | 0.006155 | 1.414284 | up |
| PA(O-16:0/18:0) | C37H75O7P | 662.52504 | 661.5178 | 10.582 | 0.006394 | 1.291354 | down |
| alpha-Tocotrienol | C29H44O2 | 424.33413 | 447.3197 | 7.12 | 0.006404 | 1.442445 | up |
| Gadenine | C30H41NO8 | 543.28322 | 542.2733 | 7.609 | 0.007024 | 1.033556 | up |
| 1,9-Nonanedithiol | C9H20S2 | 192.10064 | 193.1086 | 4.465 | 0.007497 | 1.967829 | down |
| (+)-N(6)-Hydroxyaphelandrine | C28H36N4O5 | 508.26857 | 507.2604 | 6.861 | 0.008078 | 1.02042 | up |
| 25-Methylcastasterone | C29H50O5 | 478.36583 | 477.3584 | 7.463 | 0.00811 | 1.341474 | up |
| LysoPE(18:4(6Z,9Z,12Z,15Z)/0:0) | C23H40NO7P | 473.25424 | 472.2454 | 6.379 | 0.00848 | 1.839047 | down |
| Rigidiusculamide A | C13H17NO4 | 251.11576 | 250.1081 | 5.561 | 0.008826 | 1.84796 | up |
| L-Histidine trimethylbetaine | C9H15N3O2 | 197.11643 | 220.1075 | 5.07 | 0.009307 | 1.069523 | up |
| PA(20:4(6Z,8E,10E,14Z)-2OH(5S,12R)/20:2(11Z,14Z)) | C43H73O10P | 780.49414 | 779.4898 | 7.114 | 0.012005 | 1.483969 | up |
| Deoxy phytoprostane J1 | C18H26O3 | 290.1882 | 289.1806 | 7.646 | 0.012164 | 1.358824 | down |
| Coenzyme Q2 | C19H26O4 | 318.18311 | 319.1902 | 6.723 | 0.012179 | 1.449021 | up |
| Erythratidine | C19H25NO4 | 331.17836 | 332.1884 | 6.028 | 0.013007 | 1.670361 | up |
| Isostemofoline | C22H29NO5 | 387.20457 | 386.1948 | 9.175 | 0.01303 | 1.878978 | down |
| Flustramine H | C21H29BrN2O | 404.14633 | 403.1426 | 5.674 | 0.013127 | 1.422405 | up |
| carbidopa | C10H14N2O4 | 226.09536 | 227.1026 | 2.208 | 0.014802 | 1.89481 | down |
| 8-hydroxy-13Z-octadecene-9,11-diynoic acid | C18H26O3 | 290.1882 | 291.1954 | 7.37 | 0.01523 | 1.324934 | down |
| Aucherine | C17H25NO6 | 339.16819 | 340.1751 | 5.886 | 0.015346 | 1.849723 | up |
| sn-glycero-3-Phosphoethanolamine | C5H14NO6P | 215.05588 | 214.0484 | 1.295 | 0.016254 | 1.376245 | up |
| butyrylglycine | C6H11NO3 | 145.07389 | 144.0666 | 4.854 | 0.01795 | 2.059858 | up |
| 4-Hydroxybenzyl alcohol | C7H8O2 | 124.05243 | 107.0493 | 6.014 | 0.019897 | 1.911861 | up |
| N-Acetyl-D-alloisoleucine | C8H15NO3 | 173.10519 | 172.0979 | 5.687 | 0.019975 | 1.100292 | up |
| Irbesartan | C25H28N6O | 428.23246 | 427.2268 | 7.41 | 0.020284 | 1.529707 | up |
| Fuscaxanthone H | C29H36O7 | 496.24611 | 495.2424 | 7.765 | 0.020905 | 1.679266 | up |
| Nopalinic acid | C10H18N2O6 | 262.11649 | 263.1219 | 5.374 | 0.021132 | 1.144471 | down |
| Serylvaline | C8H16N2O4 | 204.11101 | 203.1031 | 2.782 | 0.02264 | 1.41208 | up |
| Chlorbenside | C13H10Cl2S | 267.98803 | 266.9805 | 4.819 | 0.023133 | 1.694594 | up |
| Neosaxitoxin | C10H17N7O5 | 315.12912 | 316.1361 | 1.347 | 0.023163 | 1.47894 | up |
| Hispolon | C12H12O4 | 220.07356 | 203.0703 | 1.272 | 0.023944 | 1.593057 | up |
| Normelicopine | C16H13NO5 | 299.07937 | 300.0889 | 5.56 | 0.024298 | 1.206045 | up |
| Cinncassiol E | C20H30O8 | 398.19407 | 397.1866 | 5.575 | 0.024979 | 1.405458 | up |
| Dodecanyl oroxylopterocarpan | C30H40O3 | 448.29774 | 447.2879 | 10.031 | 0.025492 | 1.992212 | up |
| axinastatin 3 | C40H60N8O8 | 780.45341 | 781.4644 | 6.503 | 0.025513 | 1.460198 | up |
| Resiquimod | C17H22N4O2 | 314.17428 | 313.1656 | 5.944 | 0.02558 | 1.099011 | up |
| 3,3',5,5'-Tetrahydroxy-4-methoxystilbene | C15H14O5 | 274.08413 | 275.0918 | 5.964 | 0.027485 | 1.967368 | down |
| Corticosterone | C21H30O4 | 346.21441 | 329.2114 | 7.101 | 0.02756 | 1.707546 | up |
| Fluorenylmethyl chloroformate | C15H11ClO2 | 258.04476 | 259.054 | 6.198 | 0.028067 | 1.090237 | up |
| Flumorph | C21H22FNO4 | 371.15329 | 370.1496 | 5.403 | 0.028152 | 1.476741 | up |
| Robustol | C26H36O4 | 412.26136 | 411.2517 | 8.318 | 0.028455 | 1.216723 | up |
| PC(2:0/18:1(12Z)-O(9S,10R)) | C28H52NO9P | 577.33797 | 578.3459 | 7.257 | 0.029128 | 1.579455 | up |
| Koninginin G | C16H30O5 | 302.20932 | 301.2016 | 6.44 | 0.029326 | 1.283421 | up |
| 9-methoxy-2,2-dimethyl-6-hydro-2H-pyrano[5,6-c]quinolin-5-one | C15H15NO3 | 257.10519 | 258.1103 | 6.046 | 0.031467 | 1.580236 | up |
| Procainamide | C13H21N3O | 235.16846 | 234.1622 | 6.469 | 0.031645 | 1.11337 | up |
| 5'-N-Methylcarboxamidoadenosine | C11H14N6O4 | 294.10765 | 295.1153 | 5.362 | 0.032102 | 2.130574 | up |
| Lucidenic acid M | C27H42O6 | 462.29814 | 461.2907 | 7.068 | 0.033306 | 1.169224 | up |
| 8-HDoHE | C22H32O3 | 344.23514 | 343.2275 | 8.345 | 0.034598 | 1.013033 | down |
| (20R)-Protopanaxadiol | C30H52O3 | 460.39165 | 483.3819 | 9.983 | 0.035131 | 1.339366 | up |
| N-gamma-Glutamyl-S-allylcysteine | C11H18N2O5S | 290.09364 | 291.1007 | 5.614 | 0.035831 | 1.09522 | up |
| Isoboldine beta-N-oxide | C19H21NO5 | 343.14197 | 342.1344 | 5.912 | 0.036362 | 1.697255 | up |
| N-Acetyl-L-tyrosine | C11H13NO4 | 223.08446 | 222.0766 | 5.241 | 0.037429 | 1.132397 | up |
| 1,5-Naphthalenediamine | C10H10N2 | 158.0844 | 157.0771 | 3.534 | 0.039383 | 1.172956 | up |
| ascaroside C3 | C9H16O6 | 220.09469 | 243.0829 | 1.399 | 0.039457 | 1.362735 | down |
| S-[1-[2-(Acetyloxy)ethyl]butyl] ethanethioate | C10H18O3S | 218.09767 | 217.0905 | 5.155 | 0.040462 | 1.043624 | up |
| Polyporusterone B | C28H44O6 | 476.31379 | 475.3063 | 6.705 | 0.040473 | 1.369015 | up |
| 3-Methylcytidine | C10H15N3O5 | 257.10117 | 302.0996 | 1.43 | 0.040963 | 1.026145 | up |
| 1,2,3,4-Tetrahydroisoquinoline-3-carboxylic acid | C10H11NO2 | 177.07898 | 176.0717 | 4.75 | 0.041387 | 1.538471 | up |
| Myrtucommulone H | C41H58O10 | 710.403 | 709.4015 | 9.415 | 0.041541 | 1.811533 | down |
| Santacruzamate A | C15H22N2O3 | 278.16304 | 323.1612 | 5.787 | 0.041586 | 1.277175 | up |
| N-Acetyl-L-tryptophan | C13H14N2O3 | 246.10044 | 245.093 | 5.641 | 0.041641 | 1.84415 | up |
| 6-Methyluracil | C5H6N2O2 | 126.04293 | 125.0355 | 3.227 | 0.041767 | 1.064544 | up |
| 2-Methoxy-9-phenyl-1H-phenalen-1-one | C20H14O2 | 286.09938 | 287.109 | 1.432 | 0.041773 | 1.114098 | up |
| 13,14-seco-Stigmasta-9(11),14-dien-3alpha-ol | C29H50O | 414.38617 | 415.3932 | 10.542 | 0.042982 | 1.973696 | up |
| Homochlorcyclizine | C19H23ClN2 | 314.15498 | 315.1632 | 8.814 | 0.043034 | 1.304201 | down |
| Aferoside C | C38H60O12 | 708.40848 | 709.4139 | 7.798 | 0.044592 | 1.763554 | down |
| Chenodeoxycholic acid-3-beta-D-glucuronide | C30H48O10 | 568.32475 | 591.3193 | 6.114 | 0.046 | 1.177229 | up |
| 3beta-Hydroxy-15-[3-methylvaleryloxy]-ent-labda-7,13E-dien | C26H44O3 | 404.32904 | 403.3211 | 7.337 | 0.046149 | 1.640729 | up |
| Paniculamine | C22H35NO5 | 393.25152 | 392.2445 | 7.924 | 0.046981 | 1.528644 | up |
| rac-Acolbifene | C29H31NO4 | 457.22531 | 480.2131 | 6.236 | 0.0473 | 1.564876 | up |
| 2-Octyl-3(2H)-isothiazolone | C11H19NOS | 213.11873 | 214.1269 | 1.423 | 0.04766 | 1.332547 | down |
| Lichenin | C6H10O5 | 162.05282 | 161.0456 | 4.997 | 0.047785 | 1.498367 | down |
| 5-Hydroxyindole-2-carboxylic acid | C9H7NO3 | 177.04259 | 236.0558 | 5.074 | 0.048191 | 2.156585 | up |
| Aflatrem | C32H39NO4 | 501.28791 | 500.2842 | 7.519 | 0.048407 | 1.213424 | up |
| Benzofuroindole | C14H9NO | 207.06841 | 206.0617 | 11.84 | 0.049619 | 1.294498 | down |
| Isopimpinellin | C13H10O5 | 246.05282 | 269.0446 | 4.434 | 0.049983 | 2.115544 | up |

Table S2 The differential metabolites between the CUMS-V and CUMS group

| **Name** | **Formula** | **MolecularWeight** | **m/z** | **RT (min)** | **Pvalue** | **VIP** | **Up.Down** |
| --- | --- | --- | --- | --- | --- | --- | --- |
| 2-Hydroxy-2,6-dimethylbenzofuran-3(2H)-one | C10H10O3 | 178.063 | 179.0705 | 5.44 | 0.002375 | 1.726177 | down |
| Khusimone | C14H20O | 204.15141 | 203.1437 | 5.913 | 0.005648 | 1.91775 | down |
| Deoxycholylthreonine | C28H47NO6 | 493.34034 | 492.3327 | 7.326 | 0.005785 | 1.346961 | down |
| alpha-5-C-(3-Hydroxybutyl)-hyacinthacine A2 | C12H23NO4 | 245.16271 | 246.1702 | 5.715 | 0.006431 | 1.03254 | down |
| threo-1-(4-Hydroxy-3-methoxyphenyl)propane-1,2-diol | C10H14O4 | 198.08921 | 243.0876 | 5.374 | 0.006524 | 1.766256 | down |
| 12-Acetoxyeremophilene | C17H26O2 | 262.19328 | 263.2005 | 7.103 | 0.007291 | 1.47137 | down |
| N-[4-(1,3-benzoxazol-2-yl)phenyl]-5-chlorothiophene-2-carboxamide | C18H11ClN2O2S | 354.02298 | 353.0157 | 1.856 | 0.007861 | 1.047798 | down |
| Carbaprostacyclin-biotin | C36H60N4O5S | 660.42844 | 659.4201 | 11.474 | 0.008266 | 1.648425 | up |
| 4-Hydroxyketamine | C13H16ClNO2 | 253.08696 | 252.08 | 1.885 | 0.008301 | 1.436247 | down |
| Sterol | C17H28O | 248.21401 | 247.2064 | 7.66 | 0.008522 | 1.987077 | down |
| Heptadecatrienoic acid | C17H28O2 | 264.20893 | 265.2164 | 6.321 | 0.00873 | 1.213165 | down |
| Chamazulene | C14H16 | 184.1252 | 185.1323 | 6.84 | 0.008749 | 1.374775 | down |
| Deoxycholyltyrosine | C33H49NO6 | 555.35599 | 554.348 | 7.247 | 0.010206 | 1.182403 | down |
| 11-Oxomogroside IA1 | C36H60O9 | 636.42373 | 635.4167 | 10.567 | 0.01075 | 1.271617 | up |
| Cholesteryl glucoside | C33H56O6 | 548.40769 | 549.4134 | 10.896 | 0.011509 | 1.910314 | up |
| Phosphonoacetate | C2H5O5P | 139.98746 | 140.9957 | 0.607 | 0.013918 | 1.124362 | down |
| 5-Hydroxyindole-2-carboxylic acid | C9H7NO3 | 177.04259 | 236.0558 | 5.074 | 0.014076 | 1.167608 | down |
| Andrachamine | C15H31NO2 | 257.23548 | 258.2428 | 7.098 | 0.014361 | 1.965078 | up |
| Americine | C31H39N5O4 | 545.30021 | 546.3066 | 6.787 | 0.015343 | 1.150295 | down |
| 5-(4-Hydroxybenzyl)thiazolidine-2,4-dione | C10H9NO3S | 223.03032 | 224.0378 | 5.668 | 0.015549 | 1.690452 | down |
| Penciclovir | C10H15N5O3 | 253.11749 | 254.1262 | 5.956 | 0.015757 | 1.388077 | down |
| Demethylphylloquinone | C30H44O2 | 436.33413 | 435.3265 | 11.426 | 0.0161 | 1.68974 | up |
| Kushenol A | C25H28O5 | 408.19368 | 431.1794 | 11.079 | 0.018875 | 1.893033 | up |
| Dehydroascorbic acid | C6H6O6 | 174.01644 | 175.0231 | 2.212 | 0.019984 | 1.158086 | down |
| Phlomisone | C29H46O6 | 490.32944 | 491.3374 | 6.385 | 0.021672 | 1.4156 | down |
| Corylinal | C16H10O5 | 282.05282 | 283.0606 | 5.524 | 0.02191 | 1.326855 | down |
| gamma-Aminobutyryllysine | C10H21N3O3 | 231.15829 | 232.1672 | 5.373 | 0.022115 | 1.826405 | up |
| Guangomide A | C31H46N4O9 | 618.32648 | 617.3224 | 8.451 | 0.022315 | 1.167563 | down |
| Dibutyl adipate | C14H26O4 | 258.18311 | 259.1904 | 6.505 | 0.023913 | 1.002255 | down |
| Prohydrojasmon | C15H26O3 | 254.1882 | 277.18 | 6.625 | 0.027892 | 1.560149 | down |
| Ilexgenin A | C30H46O6 | 502.32944 | 501.322 | 6.777 | 0.029045 | 1.118457 | down |
| Dibenzyl disulfide | C14H14S2 | 246.05369 | 245.0485 | 5.844 | 0.029923 | 1.167801 | down |
| Cavinine | C18H21NO6 | 347.13689 | 348.1443 | 5.705 | 0.030237 | 1.6965 | down |
| Topsentisterol C1 | C29H46O4 | 458.33961 | 459.3471 | 7.716 | 0.030786 | 1.332841 | down |
| N-(5-cyclohexyl-1,3,4-oxadiazol-2-yl)-1H-indole-3-carboxamide | C17H18N4O2 | 310.14298 | 333.1332 | 5.409 | 0.031641 | 1.840325 | down |
| Diacetylmorphine | C21H23NO5 | 369.15762 | 370.1641 | 5.517 | 0.032339 | 1.424185 | down |
| LysoPG(16:0/0:0) | C22H45O9P | 484.28012 | 483.2688 | 8.325 | 0.032956 | 1.396814 | down |
| 3-(4-chlorophenyl)-7-hydroxy-2-methyl-4H-chromen-4-one | C16H11ClO3 | 286.03967 | 285.035 | 5.086 | 0.033805 | 1.523653 | up |
| 2-Phenylglycine | C8H9NO2 | 151.06333 | 152.0707 | 0.736 | 0.033819 | 1.363393 | down |
| 3-Methylindole | C9H9N | 131.0735 | 132.0807 | 5.288 | 0.035279 | 1.524614 | down |
| 2,5-Furandimethanol | C6H8O3 | 128.04735 | 151.0352 | 1.179 | 0.03686 | 1.113853 | down |
| (9Z,12Z)-3-hydroxyhexadecadienoylcarnitine | C23H41NO5 | 411.29847 | 410.2911 | 9.122 | 0.038177 | 1.631966 | up |
| Flumazenil acid | C13H10FN3O3 | 275.07062 | 258.0652 | 5.079 | 0.039243 | 1.76417 | down |
| Phenoxodiol | C15H12O3 | 240.07864 | 241.0863 | 5.558 | 0.041379 | 1.427487 | down |
| 1,4-Dithiane-2,5-diol | C4H8O2S2 | 151.99657 | 153.0032 | 1.176 | 0.042653 | 1.229563 | down |
| Leucine-beta-naphthylamide | C16H20N2O | 256.15756 | 255.1507 | 6.389 | 0.043901 | 1.175729 | down |
| Ethoxybenzyl diethylenetriamine | C13H23N3O | 237.18411 | 238.1915 | 5.156 | 0.043972 | 1.827248 | down |
| Xylitol | C5H12O5 | 152.06848 | 151.0613 | 1.331 | 0.044213 | 1.232375 | down |
| 4Z,7Z,10Z,13Z-hexadecatetraenoic acid | C16H24O2 | 248.17763 | 249.185 | 6.437 | 0.04529 | 1.077789 | down |
| Indolelactic acid | C11H11NO3 | 205.07389 | 188.0705 | 5.284 | 0.046202 | 1.006557 | down |
| Gamabufotalin | C24H34O5 | 402.24063 | 403.2465 | 6.399 | 0.046674 | 2.22734 | down |
| PG(20:5(5Z,8Z,11Z,14Z,16E)-OH(18)/i-14:0) | C40H69O11P | 756.45775 | 755.4434 | 8.458 | 0.049693 | 1.892479 | down |
| Pancritamine | C21H29NO5 | 375.20457 | 374.1998 | 7.903 | 0.049785 | 1.795768 | down |
